# Characterization of the Chemical Composition and Biological Activities of Bog Bilberry (*Vaccinium uliginosum* L.) Leaf Extracts Obtained via Various Extraction Techniques

**DOI:** 10.3390/foods13020258

**Published:** 2024-01-13

**Authors:** Bianca Eugenia Ștefănescu, Sonia Ancuța Socaci, Anca Corina Fărcaș, Silvia Amalia Nemeș, Bernadette Emőke Teleky, Gheorghe Adrian Martău, Lavinia Florina Călinoiu, Laura Mitrea, Floricuța Ranga, Dan Grigoroaea, Dan Cristian Vodnar, Carmen Socaciu

**Affiliations:** 1 Life Science Institute, University of Agricultural Sciences and Veterinary Medicine, 400372 Cluj-Napoca, Romania; bianca.vodnar@usamvcluj.ro (B.E.Ș.); amalia.nemes@usamvcluj.ro (S.A.N.); bernadette.teleky@usamvcluj.ro (B.E.T.); adrian.martau@usamvcluj.ro (G.A.M.); floricutza_ro@yahoo.com (F.R.); dan.vodnar@usamvcluj.ro (D.C.V.); 2 Department of Food Science, Faculty of Food Science and Technology, University of Agricultural Sciences and Veterinary Medicine, 400372 Cluj-Napoca, Romania; sonia.socaci@usamvcluj.ro (S.A.S.); anca.farcas@usamvcluj.ro (A.C.F.); carmen.socaciu@usamvcluj.ro (C.S.); 3 Department of Food Engineering, Faculty of Food Science and Technology, University of Agricultural Sciences and Veterinary Medicine, 400372 Cluj-Napoca, Romania; 4 Călimani National Park Administration, Șaru Dornei, 727515 Suceava, Romania; danranger1966@yahoo.com

**Keywords:** bog bilberry leaves, biological activities, conventional extraction, high-pressure extraction, microwave-assisted extraction, polyphenolic compounds, ultrasound-assisted extraction

## Abstract

This investigation aimed to assess the chemical composition and biological activities of bog bilberry (*Vaccinium uliginosum* L.) leaves. Hydroethanolic extracts were obtained using four extraction techniques: one conventional (CE) and three alternative methods; ultrasound (UAE), microwave (MAE) and high-pressure (HPE) extractions. Spectrophotometric analysis was conducted to determine their chemical content, including the total phenolic content (TPC) and total flavonoid content (TFC). Furthermore, their antioxidative and antimicrobial properties were evaluated. HPLC (high performance liquid chromatography) analysis identified and quantified 17 phenolic compounds, with chlorogenic acid being the predominant compound, with the lowest level (37.36 ± 0.06 mg/g) for the bog bilberry leaf extract obtained by CE and the highest levels (e.g., HPE = 44.47 ± 0.08 mg/g) for the bog bilberry leaf extracts obtained by the alternative methods. Extracts obtained by HPE, UAE and MAE presented TPC values (135.75 ± 2.86 mg GAE/g; 130.52 ± 1.99 mg GAE/g; 119.23 ± 1.79 mg GAE/g) higher than those obtained by the CE method (113.07 ± 0.98 mg GAE/g). Regarding the TFC values, similar to TPC, the highest levels were registered in the extracts obtained by alternative methods (HPE = 43.16 ± 0.12 mg QE/g; MAE = 39.79 ± 0.41 mg QE/g and UAE = 33.89 ± 0.35 mg QE/g), while the CE extract registered the lowest level, 31.47 ± 0.28 mg QE/g. In the case of DPPH (1,1-diphenyl-2-picrylhydrazyl) antioxidant activity, the extracts from HPE, UAE and MAE exhibited the strongest radical scavenging capacities of 71.14%, 63.13% and 60.84%, respectively, whereas the CE extract registered only 55.37%. According to Microbiology Reader LogPhase 600 (BioTek), a common MIC value of 8.88 mg/mL was registered for all types of extracts against *Staphylococcus aureus* (Gram-positive bacteria) and *Salmonella enterica* (Gram-negative bacteria). Moreover, the alternative extraction methods (UAE, HPE) effectively inhibited the growth of *Candida parapsilosis*, in comparison to the lack of inhibition from the CE method. This study provides valuable insights into bog bilberry leaf extracts, reporting a comprehensive evaluation of their chemical composition and associated biological activities, with alternative extraction methods presenting greater potential for the recovery of phenolic compounds with increased biological activities than the conventional method.

## 1. Introduction

Significant interest has grown in using bioactive compounds obtained from plants for the treatment and/or prevention of various non-communicable diseases. Secondary metabolites, including phenolic compounds, carotenoids, and other naturally occurring plant-derived molecules, have been the subject of numerous studies, revealing their beneficial impact on health [1]. Additionally, there has been a considerable focus on researching and developing novel plant-derived functional products and dietary supplements, characterized as nutrient-rich foods with a heightened concentration of antioxidants, a subject that has been extensively investigated in recent years [2]. Many species of the *Vaccinium* genus are renowned for containing a large amount and variety of phenolic compounds [3,4,5,6].

*Vaccinium uliginosum* L. (bog bilberry) is a wild bush indigenous to many parts of the Northern Hemisphere, particularly at higher altitudes in Asia, and in North America and Europe. This small shrub is circumpolar in the Arctic and boreal regions, and it grows on moist and acidic ground, and many different types of wildlife animals consume both the leaves and the fruits [7].

The fruits of *V. uliginosum* are characterized by the presence of anthocyanins and flavonols. Bog bilberries have a distinct flavonol and anthocyanidin profile compared to other *Vaccinium* berries. As a result, it appears that their phenolic profile could be utilized to distinguish them from other berries [6,8]. 

The leaves of *V. uliginosum* are characterized by several groups of phenolic compounds. In a study by Stanoeva et al. [9], their results revealed the presence of 20 phenolics in the leaf extract from five groups of phenolic compounds: phenolic acids, flavonols, flavanols, iridoids and cinchonain. The extract obtained from *V. uliginosum* leaves is abundant in chlorogenic acid, comprising 64% of the total amount of phenolic acids. The leaves also contain several other phenolic acids, like caffeoylquinic acid, p-coumaroylquinic acid derivatives, feruloylquinic acid, gallic acid derivatives, and various flavonols, derivatives of quercetin, kaempferol and isorhamnetin [9]. 

The plant material contains a wide range of polyphenolic structures, including simple, complex or polymerized phenolic compounds. These compounds often interact with other molecules naturally present in plants, such as polysaccharides and lipids, making the recovery of these polyphenols a challenging process. For this reason, finding an optimum extraction technique that results in the outstanding recovery of phenolic compounds from plant material is an essential stage in the research’s success, and various methods may be employed to achieve the purpose [10].

Conventional extraction (CE) methods are standard and easy to perform, although they require a significant volume of solvents and are often demanding in terms of time and energy. These methods could also lead to the deterioration of thermally sensitive polyphenolics and are frequently challenging, rendering them unsuitable for large-scale applications [10,11]. Due to these limitations, there is an increasing concern about using alternative and environmentally friendly methods for the recovery of polyphenolic compounds from plant materials. The main objective of studying alternative extraction techniques is to minimize the extraction time, decrease the amount of energy consumed and the volume of solvent, increase the extraction yield, and reduce negative environmental impact [11].

Ultrasound-assisted extraction (UAE), microwave-assisted extraction (MAE) and high-pressure extraction (HPE) have gained attention because of their multiple benefits, including increased yields of extracted compounds and reduced extraction duration and solvent utilization [12]. 

UAE is generally considered a highly productive and economical method for extracting phenolic compounds from plant-based materials. This is due to the widespread use and effectiveness of ultrasonic equipment. In addition, UAE allows the use of lower temperatures and the preservation of thermally sensitive compounds [13,14]. UAE utilizes high-frequency mechanical waves to generate the cavitation phenomenon. Cavitation is the occurrence of the development and subsequent destruction of cavities in a liquid due to the passage of ultrasonic waves, subject to certain conditions. This effect results in enhanced interaction between the solvent and the cell content, leading to the improved extraction of phenolic compounds [15,16].

The defining aspect of MAE is the interdependent interaction of the processes related to the transfer of heat and mass, in which the two gradients are acting in a single direction, and associated with the volumetric dispersal of heat within the radiated environment. In addition, it has been observed that the heating process results in interior pressure, leading cell walls to break down, facilitating the solvent’s access inside and promoting the extraction of bioactive compounds [17].

HPE is one of the developing technologies that has been effectively used to extract biologically active compounds from plant-based materials [18]. HPE induces a significant pressure gradient between the cell’s interior and exterior, inducing the structural deformation of the cell walls and membranes. This deformation increases the cells’ permeability and, consequently, increases bioactive compound extraction into the solvent [18,19].

To our knowledge, investigations on the chemical composition of Romanian bog bilberry leaves are poor and have yet to be performed. Hence, this research aimed to extract phenolic compounds from bog bilberry leaves using various extractions techniques: CE, UAE, MAE and HPE. Additionally, the study evaluated their biological activities (antioxidant and antimicrobial) along with assessing their phenolic and flavonoid contents.

## 2. Materials and Methods

### 2.1. Chemicals and Reagents

The reference substances (catechin, chlorogenic acid, quercetin and gallic acid) were bought from Sigma-Aldrich (Steinheim, Germany). The same source was used to obtain the chemical reagents needed for the relevant analytical techniques (extraction, chemical and biological characterization). The culture media for the antimicrobial activity were purchased from BioMerieux (Craponne, France) and Sigma-Aldrich (Steinheim, Germany).

### 2.2. Plant Material

The leaves of *V. uliginosum* L. were harvested in the autumn of 2021 from the spontaneous wild flora of 12 Apostoli, Suceava county, Romania, and, afterwards, the leaves were dried (7–10 days, room temperature, darkroom) as previously described in our other studies [5,20,21]. The dried leaves were ground to achieve a fine powder and stored in a dark, cool, dry place until the analyses were completed. The extraction solvent, namely ethanol/water (40% *v*/*v*) and the solid/liquid ratio adopted for all the extraction methods (1:14) were based on our previous studies [5,20], whereas in the present study we have doubled the quantity of plant material for concentration purposes, which should be reflected in the increased biological activities.

### 2.3. Extraction Procedures

#### 2.3.1. Conventional Extraction Protocol

For CE, the previously validated method by Dahmoune et al. [22] was followed, with slight modifications: over 1.5 g of plant powder was added 21 mL of ethanol/water (40%, *v*/*v*) in a glassware-type container (Erlenmeyer) that was closed during the extraction. After stirring for 2 h (750 rpm, room temperature) using magnetic stirrer equipment (Heidolph MR-Hei-Standard, Schwabach, Germany), the mixture was centrifuged at 10,000 rpm for 10 min at 24 °C, and the supernatant was filtered and stored at −18 °C until further analyses. 

#### 2.3.2. Ultrasound-Assisted Extraction Protocol

For the UAE, our previous method [5] was used, with slight modifications: the leaf powder (1.5 g) was extracted with 21 mL 40% *v*/*v* ethanol/water for 30 min in a closed glassware-type container (Erlenmeyer) using an ultrasonic bath (Elmasonic E15H, Elma, Singen, Germany) at room temperature. After centrifugation at 10,000 rpm for 10 min at 24 °C, the supernatant was filtered and stored (−18 °C) until further analyses.

#### 2.3.3. Microwave-Assisted Extraction Protocol

MAE of the phenolic compounds from bog bilberry leaves was conducted following the previous method of Nisca et al. [23], with slight modifications. Briefly, a Milestone ETHOS-X microwave oven (Milestone srl, Bergamo, Italy) system with 40% *v*/*v* ethanol/water solvent at 280 W for 5 min and a closed glassware-type container (Erlenmeyer) were used. The amount of powdered sample used for extraction was 1.5 g, along with 21 mL of extraction solvent. After the MAE, the extract was cooled at room temperature (from 85 °C), centrifuged for 10 min at 10,000 rpm, 24 °C, and the supernatant was recovered and stored at −18 °C until further analyses.

#### 2.3.4. High-Pressure Extraction Protocol 

The HPE procedure was conducted following the study of Ben Hamissa et al. [24], with slight modifications as follows: a Parr 4790 reactor (PARR Instrument Company, Moline, IL, USA) was used, outfitted with a Controller 4838 and modified with dual valves and pressure regulators to enable the controlled introduction and evacuation of CO_2_ and N_2_ gases within the reaction chamber. In two steps, 1.5 g of bog bilberry leaf powder was extracted in the static mode for 60 min using 21 mL of 40% *v*/*v* ethanol/water as a solvent. In the first stage, the experiment was carried out by replacing air by flushing carbon dioxide through the hermetically closed stainless steel chamber, before increasing and maintaining the pressure at 1000 kPa for 10 min. The second step involved introducing nitrogen until the gas mixture reached a stable pressure of 4000 kPa at 50 °C. After 50 min, the gas mixture was carefully ejected from the reaction chamber, and the extract was separated by centrifugation at 10,000 rpm for 10 min at 24 °C. The resulting supernatant was stored at a temperature of −18 °C, pending further analyses.

### 2.4. Analysis of Phenolic Compounds

#### 2.4.1. HPLC-DAD-ESI-MS Analysis 

Phenolic content identification and quantification were conducted via High Performance Liquid-Chromatography, HPLC-DAD-ESI-MS analysis, using an Agilent 1200 HPLC system equipped with a DAD detector linked to an MS-detector single-quadrupole Agilent 6110. The separation of phenolic compounds employed an Eclipse XDB C18 column (4.6 × 150 mm, particle size 5 µm) from Agilent Technologies, Santa Clara, CA, USA. Two gradients were utilized: the first comprised 0.1% acetic acid/acetonitrile (99:1) in distilled water (*v*/*v*) (solvent A), and the second contained 0.1% acetic acid in acetonitrile (*v*/*v*) (solvent B). The elution followed the procedure defined by Dulf et al. [25] at a flow rate of 0.5 mL/min. For MS fragmentation, the ESI (+) mode scanned a range of 100–1200 *m*/*z*, with the capillary voltage set at 3000 V, temperature at 350 °C and nitrogen flow at 8 L/min. DAD was utilized to measure the eluent, recording absorbance spectra (200–600 nm) throughout each run. Data examination was performed using Agilent ChemStation Software (Rev B.02.01–SR2 [260], Palo Alto, CA, USA). The identification and quantification of phenolic compounds involved comparing retention times, UV–Vis absorbance spectra, and mass spectra of peaks with three reference standards. The flavanol subclass compounds were measured using a calibration curve generated with a catechin standard within the concentration range of 10–200 µg/mL and presented as catechin equivalents (mg catechin/g plant material) (y = 15.224x − 130.24, r^2^ = 0.9985). For the hydroxycinnamic acid subclass, quantification relied on a calibration curve established using chlorogenic acid in the range of 10–50 µg/mL, denoted as chlorogenic equivalents (mg chlorogenic acid/g plant material) (y = 22.585x − 36.728, r^2^ = 0.9937). Quantification of flavonols was achieved using a calibration curve constructed using quercetin, in the concentration range of 10–200 µg/mL, expressed as quercetin equivalents (mg quercetin/g plant material) (y = 87.392x + 78.795, r^2^ = 0.9951).

#### 2.4.2. Total Phenolic Content

Total phenolic content (TPC) was assessed using the Folin–Ciocalteu method [26]. Aliquots of 25 µL of sample were mixed with 1.8 mL distilled water in a 24-well microplate. The extracts were mixed with 125 µL Folin–Ciocalteu reagent (0.2 N) and maintained at room temperature for 5 min. Afterward, the mixture was supplemented with 340 µL of a 7.5% (*w/v*) Na_2_CO_3_ solution to establish the initial conditions (pH ~ 10) facilitating the redox interaction between phenolic compounds and the Folin–Ciocalteu reagent. Subsequently, the solution was incubated in darkness at 25 °C for 2 h. A blank was prepared using ethanol, and the absorbance was read at 760 nm using a microplate reader (BioTek Instruments, Winooski, VT, USA). Gallic acid (0.01–1.00 mg/mL) was used to create the standard curve, and the TPC in the samples was recorded as gallic acid equivalent (GAE) (mg GAE/g of plant material). 

#### 2.4.3. Total Flavonoid Content

The extracts’ total flavonoid content (TFC) was assessed using the aluminum chloride colorimetric method, following the protocol described in a previously published study [27], with slight modifications. The extracts were diluted with 720 µL of distilled water, and 90 µL of 5% NaNO_2_ was added. After a 5 min incubation, the mixture was treated with 90 µL of 10% AlCl_3_, followed by an additional 5 min incubation. Next, the mixture received an addition of 600 µL of 1 N NaOH. Subsequently, the absorbance was measured at 510 nm, employing quercetin as the reference standard. Each determination was performed in triplicate. The total flavonoid content was recorded as quercetin equivalent (QE) (mg QE/g plant material).

### 2.5. DPPH Antioxidant Capacity

The antioxidant capacity of the extracts was assessed through the utilization of the DPPH (1,1-diphenyl-2-picrylhydrazyl) method for assessing free radical scavenging capacity, following the protocol described previously [28]. To assess the antioxidant activity of the samples, we created triplicate preparations by combining 35 μL of previously hydroethanol-extracted samples with 250 μL of ethanol-based DPPH solution. After incubating the solution for 30 min at room temperature, in darkness, we measured the absorbance at 515 nm using a multi-mode plate reader (BioTek, Winuschi, VT, USA). The DPPH inhibition percentage (I%) was calculated as follows: I% = [(A_0_ − A_E_)/A_0_] × 100, where A_0_ = absorbance of blank and A_E_ = absorbance of the extract.

### 2.6. Antimicrobial Activity

#### 2.6.1. Microbial Strains

The following standard microbial strains (obtained from the Food Biotechnology Laboratory, UASVM, Cluj-Napoca, Romania) were tested: *Staphylococcus aureus* subsp. *aureus* ATCC 29213, *Enterococcus faecalis* ATCC 29212, *Staphylococcus epidermidis* ATCC 12228, *Candida parapsilosis* ATCC 22019, *Candida zeylanoides* ATCC 20367, *Escherichia coli* ATCC 25922, *Pseudomonas aeruginosa* ATCC 27853 and *Salmonella enterica* (*S. typhimurium*) ATCC 14028. The strains were grown in test tubes containing 9 mL sterile TSB (tryptic soy broth), MH (Mueller–Hinton), BHI (brain heart infusion), NB (nutrient broth) and YPD (yeast extract, peptone, dextrose). The tubes with TSB were incubated for 24 h at 37 °C for *E. coli* and at 30 °C for *C. parapsilosis*. The tubes with MH were incubated for 24 h at 37 °C for *S. aureus* and *P. aeruginosa*. The tubes with BHI were incubated for 24 h at 37 °C for *E. faecalis*. The tubes with NB were incubated for 24 h at 37 °C for *S. epidermidis* and *S. enterica* (*S. typhimurium*). The tubes with YPD were incubated for 24 h at 30 °C for *C. zeylanoides*. A loopful of inoculum was transferred to a growth agar medium. Plates were incubated for 24 h at 37 °C or 30 °C, respectively. Bacterial morphology was confirmed by optical microscopy (Nikon ECLIPSE Ci-L, Tokyo, Japan) for an accurate interpretation of results and extra justifications of the bacteria response. Multiple colonies of each strain grown on the mentioned media were moved into 9 mL of sterile saline solution (8.5 g/L NaCl) and adjusted to match the turbidity of McFarland 0.5 standard (10^8^ CFU/mL). Subsequently, microbial suspensions of 10^5^ CFU/mL for bacteria and 10^6^ CFU/mL for *Candida* spp., after suitable dilution, were prepared to be added to individual wells of the microplate.

#### 2.6.2. Determination of the Minimum Inhibitory Concentration (MIC)

The MIC was determined through the resazurin microtiter plate-based antibacterial assay [29]. Initially, 100 µL of a specific sterile medium for each strain was dispensed into the wells of a 96-well microplate. Subsequently, 100 µL of each extract (71.43 mg/mL concentration) was introduced into the first well, with consecutive 2-fold dilutions prepared across each row by transferring 100 µL from well to well. The excess 100 µL in the final well of the row was removed. Following this, 10 µL of inoculum (10^5^/10^6^ CFU/mL) was added to all wells. Positive controls (C+) consisted of Gentamicin (0.4 mg/mL in saline solution) or Ketoconazole (1 mg/mL in DMSO), while the negative control (C−) involved the extraction solvent (ethanol 40%). The microplates were then incubated for 20–22 h at 37 °C or 30 °C, after which 20 µL of sterile 0.2 mg/mL resazurin aqueous solution was added to all wells. Subsequent incubation for 2 h at 37 °C or 30 °C ensued. At the end of this period, the viable bacterial cells caused the resazurin (initially blue and non-fluorescent) to oxidize into resorufin (pink and fluorescent) within the wells. Thus, the concentration in the last well of each row that retained a blue color signified the complete inhibition of bacterial growth, indicating the MIC. Each experiment was performed in triplicate.

#### 2.6.3. Determination of the Minimum Inhibitory Concentration (MIC) Using a Microbiology Reader LogPhase 600 

The MIC was determined for *Staphylococcus aureus* subsp. *aureus* ATCC 29213 and *Salmonella enterica* (*S. typhimurium*) ATCC 14028 using a Microbiology Reader LogPhase 600 (Agilent BioTek, Santa Clara, CA, USA). A volume of 100 µL of sterile medium specific to each strain was added to the wells of a 96-well microplate. Then, 100 µL of each extract (71.43 mg/mL concentration) was added in the first well, and serial 2-fold dilutions were made in the subsequent wells of each row by transferring 100 µL from well to well. The surplus of 100 µL in the last well of the row was discarded. Then, 10 µL of inoculum (10^5^ CFU/mL) was added to all the wells. Gentamicin (0.4 mg/mL in saline solution) was used as the positive control (C+), and the extraction solvent (ethanol 40%) was the negative control (C−). The microplates were incubated in the Microbiology Reader LogPhase 600 for 24 h at 37 °C and 600 rpm, and plates were read at 600 nm absorbance to determine their optical density (OD). The increase in OD versus the initial load of each microorganism added to the extracts (since these loads were not visually detected during observation) was considered a consequence of bacterial growth, indicating no antimicrobial effect. Therefore, the MIC was defined as the concentration at which no OD increase was observed in comparison with the initial loads/values [30].

### 2.7. Statistical Analysis

The outcomes of each study (each with its three or four replicates) were presented as the mean value ± SD. Statistical analysis was conducted using Graph Prism Version 8.0.1 (GraphPad Software Inc., San Diego, CA, USA) via a one-way ANOVA, followed by Tukey’s multiple comparison tests. Significant distinctions between means were considered statistically significant at a 5% significance level.

## 3. Results and Discussions

### 3.1. Phenolic Profile of Bog Bilberry Leaves under Different Extraction Methods

Seventeen phenolic compounds were detected in the leaves of the Romanian bog bilberry. They belong to three phenolic groups: hydroxycinnamic acids, flavanols and flavonols. It is essential to mention that all 17 phenolic compounds were identified in the samples obtained by all four extraction methods (Table 1). The identified phenolic acids include chlorogenic acid (5-caffeoylquinic acid), neochlorogenic acid (3-caffeoylquinic acid) and caffeic acid. Within the flavanols class, four compounds were identified: gallocatechin, epicatechin, procyanidin dimer and procyanidin trimer. Additionally, in the flavonols class, ten compounds were identified, including quercetin, kaempferol and derivatives of quercetin, kaempferol and isorhamnetin. These findings are aligned with the research of Stanoeva et al. [9], in which the bog bilberry fruits and leaves harvested from northwestern Macedonia were investigated.

Of the phenolic compounds, flavonols exhibited the highest presence in terms of number, but ranked second in terms of the highest amounts for all four extraction methods (Table 2). Moreover, hydroxycinnamic acids were the most prevalent subclass in terms of the highest levels, ranging from 68.99 ± 0.25 mg/g (in the CE extract) to 85.41 ± 0.22 mg/g (in the HPE extract). It is significant to mention that our results are in agreement with the literature [10,31]. In the studies of Dobroslavić et al. [10,31], according to their UPLC-MS/MS results, the content of phenolic acids of *Laurus nobilis* L. leaf extract obtained by pressurized liquid extraction was higher than that obtained by other extraction methods (conventional heat reflux, UAE and MAE extractions). These results may be due to the demonstrated thermal stability of phenolic acids, particularly hydroxycinnamic acids [32]. Similar results were presented in our previous study, where the extracts from blueberry leaves obtained by UAE presented the highest amounts of hydroxycinnamic acids compared to the other phenolic groups [20].

Among the identified phenolic compounds, chlorogenic acid, belonging to the hydroxycinnamic group, showed the highest concentration, with its lowest level (37.36 ± 0.06 mg/g) for the bog bilberry leaf extract obtained by CE and its highest levels (e.g., HPE = 44.47 ± 0.08 mg/g) for the bog bilberry leaf extract obtained by alternative methods. Neochlorogenic acid, also a hydroxycinnamic acid, was the second most abundant phenolic compound identified in the leaves of bog bilberries, with a concentration ranging from 24.16 ± 0.06 mg/g (for CE method) to 32.50 ± 0.04 mg/g (for UAE method). An increase in photosynthetic active radiation significantly improved the total amount of hydroxycinnamic acids, as observed by Bidel et al. [33]. To protect vital cells against damaging UV radiation, hydroxycinnamic acids will probably accumulate more when exposed to intense light [34]. In addition, the accumulation of plants’ secondary metabolites, specifically hydroxycinnamic acids, is sustained at higher altitudes and cooler temperatures [35]. Regarding caffeic acid, the extract obtained by HPE had a significant concentration (9.87 ± 0.07 mg/g).

Interestingly, in the research of Stanoeva et al. [9], similar to our results, chlorogenic acid was the predominant compound found in the leaves of bog bilberry, representing 64% of the total phenolic acid derivatives. However, they did not report the presence of neochlorogenic and caffeic acids in their extract from bog bilberry leaves. To our knowledge, there have been no reports of these compounds’ occurrence in bog bilberry leaves, only in the fruits and leaves of other *Vaccinium* species [3,20,21,36].

Considering the flavonols class, quercetin-glucuronide was the predominant phenolic compound measured, for which the extracts obtained by alternative methods (e.g., HPE, MAE) registered the highest concentrations (16.09 ± 0.04 mg/g; 13.65 ± 0.04 mg/g) in comparison to CE (13.54 ± 0.04 mg/g). The second most abundant flavonol identified in the bog bilberry extracts was kaempferol-glucuronide, where again the modern techniques registered the most significant quantities: HPE = 10.35 ± 0.15 mg/g and UAE = 9.22 ± 0.11 mg/g, in comparison with CE (8.71 ± 0.07 mg/g). Traces of quercetin-rutinoside (Rutin) were found in all the extracts. In the study of Stanoeva et al. [9], quercetin-rutinoside (Rutin) was not detected. However, in our previous studies [5,20] on other *Vaccinium* spp. leaves, quercetin-rutinoside (Rutin) quantities were 50 to 70-fold higher than in the bog bilberry leaves analyzed in the present study. 

Moreover, quercetin and kaempferol aglycones were also quantified in small amounts. These flavonol aglycones were not detected in the research of Stanoeva et al. [9], where bog bilberry leaves were analyzed, but they have been quantified in other *Vaccinium* spp. leaves [3,4,5,20]. Additionally, two glycosides of isorhamnetin were identified in bog bilberry leaves: isorhamnetin-glucuronide and isorhamnetin-rhamnoside. At the same time, the study of Stanoeva et al. [9] reported only one glycoside of isorhamnetin. As previously reported [3,4,9], and similar to our results, kaempferol glycosides were considerably less prevalent than quercetin glycosides in the *Vaccinium* spp.

In the flavanols group, four compounds were detected, with gallocatechin as the dominant phenolic compound, with its highest concentration found in the extract obtained by the modern method HPE, namely 11.23 ± 0.09 mg/g. Epicatechin was identified in all four extracts, ranging from 5.15 ± 0.05 mg/g to 7.69 ± 0.05 mg/g. The two procyanidins were detected in all the extracts, with levels comparable to the amount of epicatechin. Stanoeva et al. [9] reported that in the leaves of bog bilberry from Macedonia, there was only one compound from the flavanol group, namely procyanidin dimer.

Significant selectivity was not induced by our extraction techniques, as indicated by the absence of noticeable variations in the characteristics of the extracts in their HPLC profiles. The extracts acquired using all four extraction methods displayed equivalent qualitative contents, respectively; the same 17 phenolic compounds were identified in all the extracts.

According to previous research [37], utilizing various types of energy, such as ultrasound, microwaves and high pressure, may provide a potentially beneficial alternative for enhancing the levels of phenolic extraction. For example, the results reported by Caldas et al. [37] in extracting phenolic compounds from grape skin showed that the UAE and MAE methods provided greater phenolic recovery within a reduced time period. 

Additionally, in the research study of Mróz et al. [38] all of the alternative extraction techniques investigated, MAE, UAE and HPE, enhanced the total recovery of phytochemicals from the flowering aerial parts of *Sideritis scardica* and *Sideritis raeseri*. 

Quantitatively, in the present study, it was observed that the highest concentrations were obtained in the bog bilberry leaf extracts obtained by modern techniques (HPE, UAE, MAE) for almost all phenolic compounds, in comparison to the extract obtained by CE. Given that distinct extraction mechanisms are employed in the HPE, UAE and MAE methods, it is appropriate to expect diverse secondary metabolites to be collected from the bog bilberry leaves when utilizing these extractions techniques. Furthermore, these extraction methods will likely uniquely impact the amounts of the individual phenolic compounds extracted [12]. 

### 3.2. Total Phenolic and Total Flavonoid Content

The TPC and TFC values of the bog bilberry leaf extracts obtained by various extraction methods are presented in Table 3. Our initial remark concerns the variation in the levels of phenolic and flavonoid constituents. In all the extracts obtained, the levels of TPC were consistently higher than those of TFC, independent of the extraction technique utilized.

Previous research studies have noted a wide range of variations in TPC for extracts derived from various *Vaccinium* species’ leaves. In our previous study [5], we observed variations in the TPC of the extracts from bilberry (*Vaccinium myrtilus* L.) leaves collected from three different habitats; the values varied from 132.47 to 135.8 mg GAE/g plant material. In the same study [5], we reported comparable TPC values in the extracts from lingonberries (*Vaccinium vitis-idaea* L.) leaves collected from three distinct habitats. Moreover, in another study on bilberry leaves [39], the reported values of the TPC were higher than our results, with values varying between 196.48 and 280.69 mg GAE/g extract. In Bujor et al.’s study [4], the TPC values of lingonberry leaf extracts were from 135 to 158 mg GAE/g dry extract, depending on the harvest period. Furthermore, in blueberry leaf (*Vaccinium corymbosum* L.) extracts the values of the TPC ranged from 98.00 to 135.55 mg GAE/g plant material, depending on the cultivar [20]. 

In the research study of Páscoa et al. [40], an extract from winter leaves of the blueberry (*V. corymbosum* L.) cultivar Aurora contained the highest TPC (227.4 mg GAE/g dry leaf) of all harvest seasons (spring, autumn and winter). Conversely, their extract from the blueberry cultivar Huron (autumn leaves) exhibited the lowest TPC (39.6 mg GAE/g dry leaf). Within the research study of Gao et al. [41], the extract obtained using 80% ethanol from the leaves of *Vaccinium dunalianum* presented a TPC value of 257.11 mg GAE/g dry extract. Our present findings exhibited a strong similarity to those reported by the above-mentioned studies, despite the utilization of different extraction methods and solvents, *Vacciunium* species, harvest seasons and geographic regions.

The extracts obtained by HPE, UAE and MAE presented TPC values (135.75 ± 2.86 mg GAE/g; 130.52 ± 1.99 mg GAE/g; 119.23 ± 1.79 mg GAE/g) higher than those obtained by the CE method (113.07 ± 0.98 mg GAE/g).

Additionally, a high efficacy of non-conventional extraction methods has been observed for a variety of plant material. For instance, in the research study of Cheng et al. [42], the TPC values of the water and 60% methanol extracts obtained from jackfruit pulp using a UAE method were high. In the same study, they reported a higher TPC of the 60% ethanol extract obtained by MAE. Similar results were reported by Routray et al. [43], where their blueberry leaf extracts obtained by UAE and MAE presented high TPC values. Analogous results were reported by Alexandre et al. [44], in which the TPC value of the extract obtained by HPE from prickly pear peel was quite significant. 

Regarding the TFC values, similar to TPC, the highest levels were registered in the extracts obtained by alternative methods (HPE = 43.16 ± 0.12 mg QE/g; MAE = 39.79 ± 0.41 mg QE/g; and UAE = 33.89 ± 0.35 mg QE/g), while the CE extract registered the lowest level, 31.47 ± 0.28 mg QE/g. These values are consistent with our previous results (31.36–67.88 mg QE/g plant material) reported for the 40% ethanol extracts of blueberry leaves obtained by UAE [20]. Moreover, our results are higher than Brezoiu et al.’s previous results (2.20–10.36 mg QE/g plant) [39] for bilberry leaves obtained by CE and UAE using ethanol or 50% ethanol. Additionally, using 80% ethanol and UAE, Gao et al. [41] reported higher values of the TFC in the leaves of *V. dunalianum*. These differences between TFC values were likely related to the composition of the solvent, the different parameters of the extraction techniques and the species of plants utilized. It is relevant to highlight that while earlier research has produced berry leaf extracts with increased or decreased TPCs and TFCs, the plant’s location and surrounding factors affect the polyphenolic composition of the leaves. It has been indicated that the temperature’s limiting effect on photosynthesis results in nearly two times higher TPC concentrations in the leaves of bilberry bushes growing in high-light locations, at higher latitudes, and/or at higher altitudes than in those growing at lower latitudes or altitudes. Furthermore, depending on biotic and abiotic pressures, there are seasonal fluctuations in the quantity and variety of phenolic compounds and the berry leaves’ antioxidant activity [4,34,36,45,46]. 

### 3.3. DPPH Antioxidant Capacity

The DPPH radical scavenging capacity of extracts obtained from the leaves of bog bilberry by various extraction techniques was assessed in order to determine their antioxidant activity. The results (Table 3) indicated statistically significant differences in the antioxidant activity of the extracts derived from non-conventional extraction in comparison with those from the CE. According to the DPPH assay, the extracts from HPE, UAE and MAE exhibited a strongest radical scavenging capacity of 71.14%, 63.13% and 60.84%, respectively, whereas the CE extract registered only 55.37%. 

Previous research studies have shown a notable direct relationship between overall phenolic and flavonoid contents and antioxidant activity [41,42,47,48]. These findings indicate that the antioxidant activity observed in bog bilberry leaf extracts could be attributed to their higher levels of TPC and TFC. As a consequence, in our previous study [20], the extracts obtained from the Toro, Elliot and Nelson varieties’ leaves (*V. corymbosum* L.), which revealed a higher polyphenolic content, displayed the greatest antioxidant activity, expressed as a percentage of inhibition (70.41%, 68.42% and 58.69%, respectively). Brezoiu et al. [39] reported comparable results in their extracts obtained from bilberry leaves; the antioxidant activity increased with the increase in the TPC values. 

The DPPH test revealed different results for bog bilberry leaf extracts obtained by various extraction techniques, and this may be explained by the different amounts of polyphenols with dihydroxyphenyl moieties in each extract, considering the different extraction parameters. The results of the HPLC revealed that the extracts from bog bilberry leaves contain phenolic acids and derivatives, including chlorogenic acid, which is known for its antioxidant properties. The antioxidant properties of these compounds emanate from o-diphenolic functionality and the presence of hydroxyl groups within their molecular structure. According to these characteristics, the molecule can donate electrons and hydrogen atoms [49]. Additionally, quercetin and quercetin derivatives, as well as other flavonols, exhibit the capacity to counteract free radicals due to the hydroxyl groups that constitute the molecule [50]. Moreover, among the polyphenolic compounds, the flavanol group as a whole, and the proanthocyanidin (procyanidin dimer and trimer) subgroup, equally possess the highest antioxidant activity [51] because of the catechol structures present in those molecules, linked by C3-OH and C4-C8 bonds, which greatly decrease the production of free radicals [52].

### 3.4. Antimicrobial Activity of Bog Bilberry Leaf Extracts

All the bog bilberry leaf extracts have been tested for their antimicrobial activity against three Gram-positive and three Gram-negative bacterial strains and against two fungi. The results of the minimum inhibitory concentration are presented in Table 4.

Although the bog bilberry leaf extracts showed antioxidant activity and presented high TPC and TFC values, the extracts displayed antimicrobial activity only against some of the tested strains. 

Regarding Gram-positive bacteria, all the extracts exhibited the same MIC towards *S. aureus*, respectively, 17.75 mg/mL. This result is in agreement with a previous research paper [53] evaluating the antibacterial activity of *Annona cherimola* phytochemicals obtained by UAE and comparing it to CE methods (maceration-MAC and Soxhlet-SE), where all the extracts presented an antimicrobial effect against *S. aureus*, with higher inhibition percentages from the UAE samples. In the present study, the strain *E. faecalis* was the most resistant. The results showed no inhibitory effect against this strain. In our previous study [20], *E. faecalis* was the most resistant strain towards all the blueberry leaf extracts tested. Regarding the *S. epidermidis* strain, only the extracts obtained by UAE and CE registered antimicrobial activity, with a MIC of 17.75 mg/mL. Our results are in line with previous studies. For example, in the study of Saifullah et al. [54], the antibacterial properties of their extracts prepared from modern techniques (MAE, UAE) and CE (SWB–shaking water bath) were not significantly different, a fact that could be due to the similarity of the phenolic compounds and antioxidant properties in the extracts obtained from these extraction techniques. In another study [55] evaluating the efficacy of two methods (agitation as the CE and UAE as the modern technique) at extracting phenolic compounds from 15 native plants, a greater inhibition capacity was obtained through UAE against three of the six bacteria studied: *Listeria monocytogenes*, *Listeria innocua* and *Salmonella choleraesuis*, whereas against the other three bacteria, *S. aureus*, *Bacillus cereus* and *E. coli*, the CE method proved to have a better antimicrobial capacity, therefore underlying the complex relationship between phenolic composition and biological activity. In the research paper of Mašković et al. [56], *Satureja hortensis* L. (summer savory) herb extracts were prepared using CE (MAC and SE) and non-conventional (UAE, MAE and subcritical water extraction-SWE) techniques. The antibacterial activity of their extracts was determined against 15 selected bacterial strains and the results showed MIC values of 7.81 μg/mL, with the SE extract having exhibited the greatest activity towards *S. aureus* and *E. coli*, the MAC extract toward *Enterobacter aerogenes* and the SWE extract toward *Staphylococcus saprophyticus*. On the other hand, the most resistant bacterial strain was *Salmonella enteritidis*. A similar research study [57], dealing with the application of CE methods (MAC and SE) and non-conventional (UAE, MAE and SWE) methods for the isolation of bioactive compounds from *Erica carnea* L. (spring heath), reported that, generally, the best antibacterial result was from the UAE extract, while the MAE and SWE extracts exhibited similar activities. The strongest activity was exerted by the SE extract against *E. coli*, the MAC extract against *E. aerogenes* and *Proteus mirabilis*, the UAE extract against *S. typhimurium* and the SWE extract against *S. saprophyticus,* with a MIC value of 7.81 μg/mL. In the research study of Gutiérrez-Sánchez et al. [58], similar findings to ours were reported regarding the lack of antimicrobial activity against specific strains. They assessed the antimicrobial capacity of their samples from the leaves of *Hamelia patens* against several strains and their results showed no inhibitory effect on the majority of studied Gram-negative and Gram-positive bacterial strains. They reported that 70% dimethyl sulfoxide was used to extract the phenolic compounds in their research study and not 70% ethanol, as was the case in another studies, whereas an explanation of the lack of antimicrobial activity from the extracts could be related to the solvent utilized [58]. 

When it comes to Gram-negative bacteria, the strain *S. enterica* was the only bacteria sensitive to the extracts, with a MIC of 8.88 mg/mL for the extract obtained by UAE and a MIC of 17.75 mg/mL for the extracts obtained by CE, MAE and HPE. The extracts displayed no antimicrobial activity against *E. coli* and *P. aeruginosa*. These results are comparable to the previous study [44], which compared the antimicrobial activity of prickly pear peel compounds extracted with modern (HPE and OM-ohmic heating) and CE (SE) techniques, whose results showed that for *S. aureus* and *S. enteritidis* the MIC obtained was 125 mg/mL, independent of the extraction method, while for *B. cereus* only the HPE extract exhibited an antimicrobial effect. Moreover, in the paper by Tanase et al. [59], the antibacterial activity of spruce bark (*Picea abies* L.) extracts obtained via CE and UAE methods was tested and the results revealed that both types of extracts had a stronger antimicrobial effect against Gram-positive cocci (*S. aureus*) compared to Gram-negative bacilli (*Klebsiella pneumoniae*, *P. aeruginosa*). However, the UAE extract presented a bactericidal effect on *K. pneumoniae* and *P. aeruginosa* while the CE extract presented a bactericidal effect only on *P. aeruginosa*. Recently, Vilkickyte et al. [60] reported that an extract from lingonberry leaves showed no antimicrobial activity against *E. coli*. Moreover, Tian et al. [61] reported that *E. coli* presented low sensitivity to an extract of berry plants and no inhibitory effect was noticed in extracts derived from bilberry, chokeberry and nettle leaves. Additionally, Silva et al. [62] observed that *E. coli* and *P. aeruginosa* were resistant to a blueberry leaf extract, and, more precisely, they did not identify any inhibition against these strains. Several previous studies reported the antimicrobial activity of other berry leaves against *E. coli* and *P. aeruginosa*. Bilberry and lingonberry leaf extracts displayed antimicrobial activity against *E. coli* and *P. aeruginosa*; these strains were the most resistant bacteria [5]. Similarly, in the research of Gil-Martínez et al. [46], *E. coli*, *P. aeruginosa*, *S. enterica* and *Shigella sonnei* were more resistant to bilberry leaf extract than other bacterial strains.

Regarding the two fungi tested in this study, none of the extracts had any effect against *C. zeylanoides*. Moreover, towards *C. parapsilosis*, only the extracts obtained by alternative extraction methods had an antimicrobial activity, with a MIC of 8.88 mg/mL.

The antimicrobial properties of plant-based extracts are often related to several constituents, including phenolic acids, flavonoids, tannins, alkaloids, terpenoids and lactones. Previous research has demonstrated that the phenolic compounds found in plants play an essential role in their antimicrobial properties. The efficacy of these properties is influenced by the particular mechanism of action of polyphenols, the amount of phenolic compounds, and the techniques used for extraction [63,64,65]. Moreover, a variety of mechanisms of action, including cytoplasmic membrane destabilization, plasma membrane permeabilization, the suppression of external microbial enzymes, direct effects on the metabolism of microbial cells and the deprivation of a substrate essential to microbial growth, are involved in the inhibition of the proliferation of bacteria [66]. Phenolic compounds are thought to affect the cytoplasmic membrane as their primary antibacterial mechanism. However, the external lipid membrane of Gram-negative bacteria may serve as an adjuvant protective barrier, which could explain why phenolic compounds are ineffective against them [20,62]. The bacteria can be more sensitive or more resistant to the action of the plant extracts.

**Figure 1:**
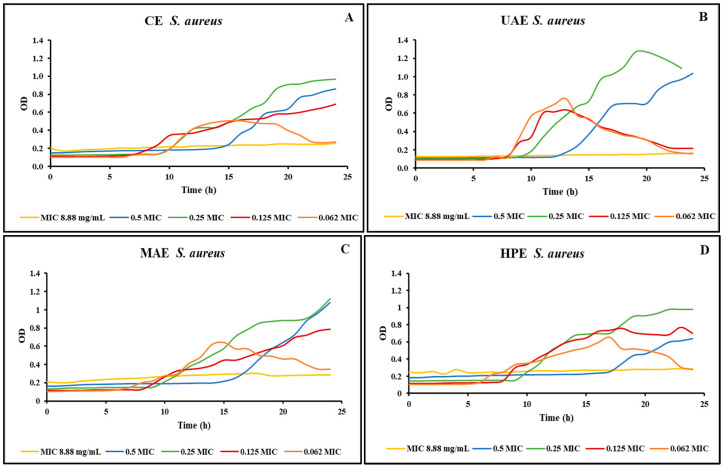
*S. aureus* growth curves for the extracts obtained by (**A**) CE, (**B**) UAE, (**C**) MAE, (**D**) HPE.

Growth curves were created, after determining the minimum inhibitory concentrations, using a Microbiology Reader LogPhase 600 (Agilent BioTek, Santa Clara, CA, USA) to improve our comprehension of the extracts’ impact on the inhibited microorganisms. The growth curves were created only for two of the microorganisms tested, because they were the only ones sensitive to all the extracts obtained from the bog bilberry leaves. Figure 1 shows the growth curves realized for the *S. aureus* strain using different concentrations of the four extracts obtained from bog bilberry leaves. Visual observation was employed first in our study to determine all MICs. However, it is not more precise than the spectrophotometric method used to create the growth curves. When microbial loads are low, cellular growth can occasionally produce turbidity invisible to the human eye, but it can be identified spectrophotometrically [30]. This situation was observed in our study. Using a resazurin microtiter plate-based antibacterial assay, the MIC for all the extracts of bog bilberry leaves was 17.75 mg/mL. 

On the other hand, as can be seen in Figure 1 with the use of a Microbiology Reader LogPhase 600 (BioTek), the MIC for the extracts was lower, 8.88 mg/mL. Additionally, it can be noticed that for lower concentrations the growth of the *S. aureus* was inhibited for a period of time. Afterward, the bacteria started to grow.

**Figure 2:**
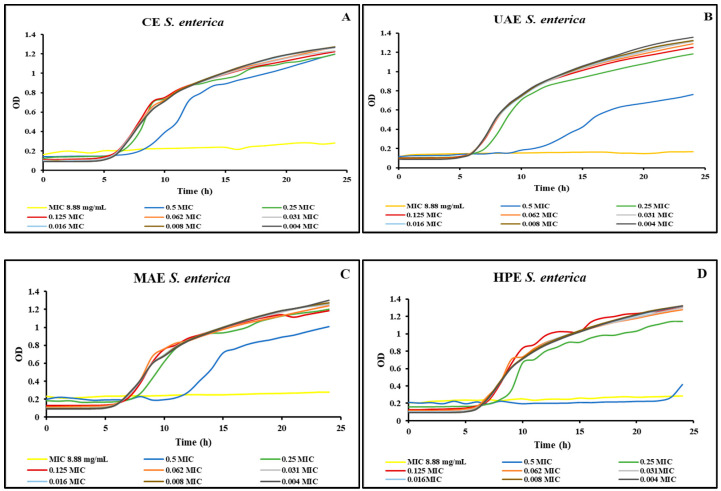
*S. enterica* growth curves for the extracts obtained by (**A**) CE, (**B**) UAE, (**C**) MAE, (**D**) HPE.

Figure 2 shows the growth curves realized for the *S. enterica* strain using different concentrations of the four extracts obtained from bog bilberry leaves. Similar to the *S. aureus* strain, for all extracts, in the case of *S. enterica* strain, the MIC was lower when using the Microbiology Reader LogPhase 600.

## 4. Conclusions

In conclusion, our study identified a rich profile of phenolic compounds in the leaves of the Romanian bog bilberry, belonging to hydroxycinnamic acids, flavanols and flavonols. Remarkably, all 17 phenolic compounds were consistently detected across all four extraction methods. The predominant phenolic acids were chlorogenic acid, neochlorogenic acid and caffeic acid, while for the flavonols class, quercetin-glucuronide was the most abundant; all in the highest quantities in the HPE, UAE and MAE extracts, and lowest in the CE-derived extract. Hydroxycinnamic acids, especially chlorogenic acid, were the most prevalent subclass across all the extraction methods, with their highest levels in alternative extraction-derived samples.

Quantitatively, HPE, UAE and MAE consistently yielded a significant phenolic and flavonoid content, along with a high antioxidant capacity, reinforcing the potential benefits of the alternative extraction techniques in comparison to conventional methods. However, antimicrobial activity was observed selectively against Gram-positive bacteria and *S. enterica*, underlining the complex relationship between phenolic composition and biological activity.

This comprehensive analysis provides valuable insights into the phenolic composition, extraction efficiency and bioactivity of *Vaccinium uliginosum* L., offering perspectives for future works (e.g., identification of the phenolic compounds/phenolic class responsible for the antibacterial activity) and supporting the potential uses and applications of these extracts in real food systems, such as functional foods and pharmaceuticals.

## Figures and Tables

**Table 1:** The phenolic compounds detected in the leaf extracts of the bog bilberry using HPLC.

Peak No.	Retention Time R_t_(min)	UV λ_max_(nm)	[M + H]^+^ (*m*/*z*)	Compound	Subclass
1	3.16	279	307, 290	Gallocatechin	Flavanol
2	11.52	330	355, 163	3-Caffeoylquinic acid (Neochlorogenic acid)	Hydroxycinnamic acid
3	12.27	330	355, 163	5-Caffeoylquinic acid (Chlorogenic acid)	Hydroxycinnamic acid
4	13.56	280	579, 291	Procyanidin dimer	Flavanol
5	13.80	282, 329	181, 163	Caffeic acid	Hydroxycinnamic acid
6	14.01	280	291	Epicatechin	Flavanol
7	14.43	280	867, 291	Procyanidin trimer	Flavanol
8	15.78	263, 355	611, 303	Quercetin-rutinoside (Rutin)	Flavonol
9	16.20	263, 355	465, 303	Quercetin-glucoside	Flavonol
10	16.44	263, 355	479, 303	Quercetin-glucuronide	Flavonol
11	17.24	263, 355	435, 303	Quercetin-arabinoside	Flavonol
12	17.43	260, 340	463, 287	Kaempferol-glucuronide	Flavonol
13	17.77	260, 360	493, 317	Isorhamnetin-glucuronide	Flavonol
14	18.44	260, 360	463, 317	Isorhamnetin-rhamnoside	Flavonol
15	18.79	260, 340	419, 287	Kaempferol-arabinoside	Flavonol
16	21.79	261, 355	303	Quercetin	Flavonol
17	23.39	260, 340	287	Kaempferol	Flavonol

**Table 2:** The concentration of individual phenolic compounds in the extracts of the bog bilberry leaves using different extraction methods, expressed as mg/g.

Phenolic Compounds		Extraction Methods			
		CE	UAE	MAE	HPE
Hydroxycinnamic acids	Neochlorogenic acid	24.16 ± 0.06	32.50 ± 0.04 ***	27.12 ± 0.08 ***	31.07 ± 0.07 ***
	Chlorogenic acid	37.36 ± 0.06	43.22 ± 0.08 ***	39.65 ± 0.01 ***	44.47 ± 0.08 ***
	Caffeic acid	7.47 ± 0.13	8.36 ± 0.08 ***	7.32 ± 0.08 *	9.87 ± 0.07 ***
Flavanols	Gallocatechin	9.92 ± 0.03	7.67 ± 0.02 ***	9.75 ± 0.07 ***	11.23 ± 0.09 ***
	Epicatechin	5.80 ± 0.05	5.93 ± 0.04 ^N.S.^	7.69 ± 0.05 ***	5.15 ± 0.05 ***
	Procyanidin dimer	4.59 ± 0.03	4.58 ± 0.02 ^N.S.^	4.86 ± 0.03 ***	3.22 ± 0.09 ***
	Procyanidin trimer	5.54 ± 0.03	4.73 ± 0.03 ***	6.16 ± 0.04 ***	4.44 ± 0.04 ***
Flavonols	Quercetin-rutinoside (Rutin)	0.37 ± 0.02	0.26 ± 0.02 ***	0.45 ± 0.02 ***	0.69 ± 0.03 ***
	Quercetin-glucoside	9.37 ± 0.03	9.07 ± 0.05 ***	9.36 ± 0.07 ^N.S.^	9.78 ± 0.04 ***
	Quercetin-glucuronide	13.54 ± 0.04	13.49 ± 0.05 ^N.S.^	13.65 ± 0.04 **	16.09 ± 0.04 ***
	Quercetin-arabinoside	1.84 ± 0.05	1.92 ± 0.02 ***	1.75 ± 0.01 ***	2.38 ± 0.02 ***
	Kaempferol-glucuronide	8.71 ± 0.07	9.22 ± 0.11 ***	8.70 ± 0.11 ^N.S.^	10.35 ± 0.15 ***
	Isorhamnetin-glucuronide	3.27 ± 0.14	3.81 ± 0.11 ***	3.25 ± 0.08 ^N.S.^	4.533 ± 0.076 ***
	Isorhamnetin-rhamnoside	0.27 ± 0.01	0.28 ± 0.0.1 **	0.34 ± 0.02 ***	0.93 ± 0.02 ***
	Kaempferol-arabinoside	0.24 ± 0.01	0.25 ± 0.02 ^N.S.^	0.29 ± 0.02 ***	0.58 ± 0.02 ***
	Quercetin	0.62 ± 0.02	0.51 ± 0.02 ***	0.39 ± 0.01 ***	0.70 ± 0.02 ***
	Kaempferol	0.41 ± 0.02	0.47 ± 0.03 ***	0.14 ± 0.01 ***	0.49 ± 0.01 ***

Phenolic compounds, including flavonoids such as flavanols and flavonols, as well as hydroxycinnamic acids, were quantified as a concentration of miligrams per gram (mg/g). The experiments were replicated four times, and the reported values represent the average and standard deviation (SD) of these replicates. Data normality was assessed using the Shapiro–Wilk test, where a *p*-value greater than 0.05 indicated normally distributed data. The mean ± SD is presented in the descriptive statistics table. To investigate significant differences between the four extraction methods for each compound, a two-way ANOVA was conducted, followed by Tukey’s multiple comparisons test. In this analysis, the second column compares CE and UAE, the third column compares CE and MAE, and the fourth column assesses the distinctions between CE and HPE. Significance levels are denoted using the following symbols: *** *p* < 0.001, ** *p* < 0.01, * *p* < 0.05, and N.S. (not significant).

**Table 3:** Total phenolic content, total flavonoid content and DPPH activity of the extracts.

Extraction Methods	TPC (mg GAE/g Plant Material)	TFC (mg QE/g Plant Material)	DPPH (I%)
CE	113.07 ± 0.98	31.47 ± 0.28	55.37%
UAE	130.52 ± 1.99 ***	33.89 ± 0.35 *	63.13%
MAE	119.23 ± 1.79 ***	39.79 ± 0.41 ***	60.84%
HPE	135.75 ± 2.86 **	43.16 ± 0.12 ***	71.14%

This study reported significance levels as *** *p* < 0.001, ** *p* < 0.01, * *p* < 0.05. Results, presented as mean ± standard deviation, were derived from three replicates. Two-way ANOVA followed by Turkey’s multiple comparisons test explored differences between the four extraction methods.

**Table 4:** The results of the determination of the minimum inhibitory concentration (MIC) (mg/mL) of the extracts against *Staphylococcus aureus* subsp. *aureus* ATCC 29213, *Enterococcus faecalis* ATCC 29212, *Staphylococcus epidermidis* ATCC 12228, *Candida parapsilosis* ATCC 22019, *Candida zeylanoides* ATCC 20367, *Escherichia coli* ATCC 25922, *Pseudomonas aeruginosa* ATCC 27853 and *Salmonella enterica* (*S. typhimurium*) ATCC 14028.

Extraction Methods	Gram (+) Bacteria			Fungi		Gram (−) Bacteria		
	*S. aureus*	*E. faecalis*	*S. epidermidis*	*C. parapsilosis*	*C. zeylanoides*	*E. coli*	*P. aeruginosa*	*S. enterica*
CE	17.75	h.c	17.75	h.c	h.c	h.c	h.c	17.75
UAE	17.75	h.c	17.75	8.88	h.c	h.c	h.c	8.88
MAE	17.75	h.c	h.c	h.c	h.c	h.c	h.c	17.75
HPE	17.75	h.c	h.c	8.88	h.c	h.c	h.c	17.75
Gentamicin	0.0001	0.013	0.002	-	-	0.003	0.0001	0.002
Ketoconazole	-	-	-	0.016	0.063	-	-	-

h.c—higher than the highest concentration tested (≤71.43 mg/mL); (-)—not tested.

## Data Availability

The data presented in this study are available on request from the corresponding author.
